# Peculiar Neurological Examination Secondary to Persistent Primitive Hypoglossal Artery

**DOI:** 10.7759/cureus.42249

**Published:** 2023-07-21

**Authors:** Haroutiun Hamzoian, Brittany Harris, Mekdes Ditamo, Shuchi Chaudhary

**Affiliations:** 1 Neurology, Orlando Regional Medical Center, Orlando, USA; 2 Vascular Neurology, Orlando Regional Medical Center, Orlando, USA

**Keywords:** vertebral artery (va), vertebral artery, digital subtraction angiography(dsa), neurological exam, anatomical variability, stroke, brain thrombectomy, vascular neurosurgery, neurology and critical care, neurology

## Abstract

A persistent primitive hypoglossal artery (PPHA) is an anatomical variant resulting in persistent carotid-vertebrobasilar anastomoses. This variant arises from the distal cervical segment of the internal carotid artery (ICA) between C1 and C3 and passes through an enlarged hypoglossal canal to join the basilar circulation. This case report describes a 60-year-old male with an acute ischemic event secondary to an occlusion in the right ICA and PPHA, resulting in a unique physical examination. Digital subtraction angiography (DSA) was utilized to visualize occlusion of the right common carotid artery with no residual right internal carotid artery or right vertebral artery filling. The patient's presenting symptoms yielded a unique neurological examination, making it difficult to localize a solitary lesion, which would account for the patient’s acute presentation. In retrospect, with angiography revealing a right PPHA, his presentation fit more thoroughly with the clinical picture. With the increased utility of neuro-endovascular procedures, clinicians have a higher probability of encountering diverse angiographical findings. With this case report, we would like to familiarize practitioners with the presence of PPHA, present unique imaging findings involving typically isolated vascular territories, and stress the importance of clinical judgment when making decisions regarding stroke care.

## Introduction

A persistent primitive hypoglossal artery (PPHA) is an anatomical variant resulting in persistent carotid-vertebrobasilar anastomoses. This variant arises from the distal cervical segment of the internal carotid artery (ICA) between C1 and C3 and passes through an enlarged hypoglossal canal to join the basilar circulation [1]. It was first described by Batujeff in 1899 on a pathological specimen; with the widespread use of angiographic imaging, it has been identified with a prevalence of 0.03% to 0.09%. PPHA is usually incidental and asymptomatic, although there have been reports describing aneurysmal sequelae along the artery’s trajectory [2]. The literature revealed intraluminal support stent-assisted coiling being safely utilized in the management of aneurysmal complications, but as acute cases of thrombotic events have not been commonly reported, no current guidelines exist for the management of ischemic complications with an underlying PPHA [3]. This case report describes a 60-year-old male with an acute ischemic event secondary to an occlusion in the right ICA and PPHA presenting with a unique physical examination.

## Case presentation

**Figure 1:**
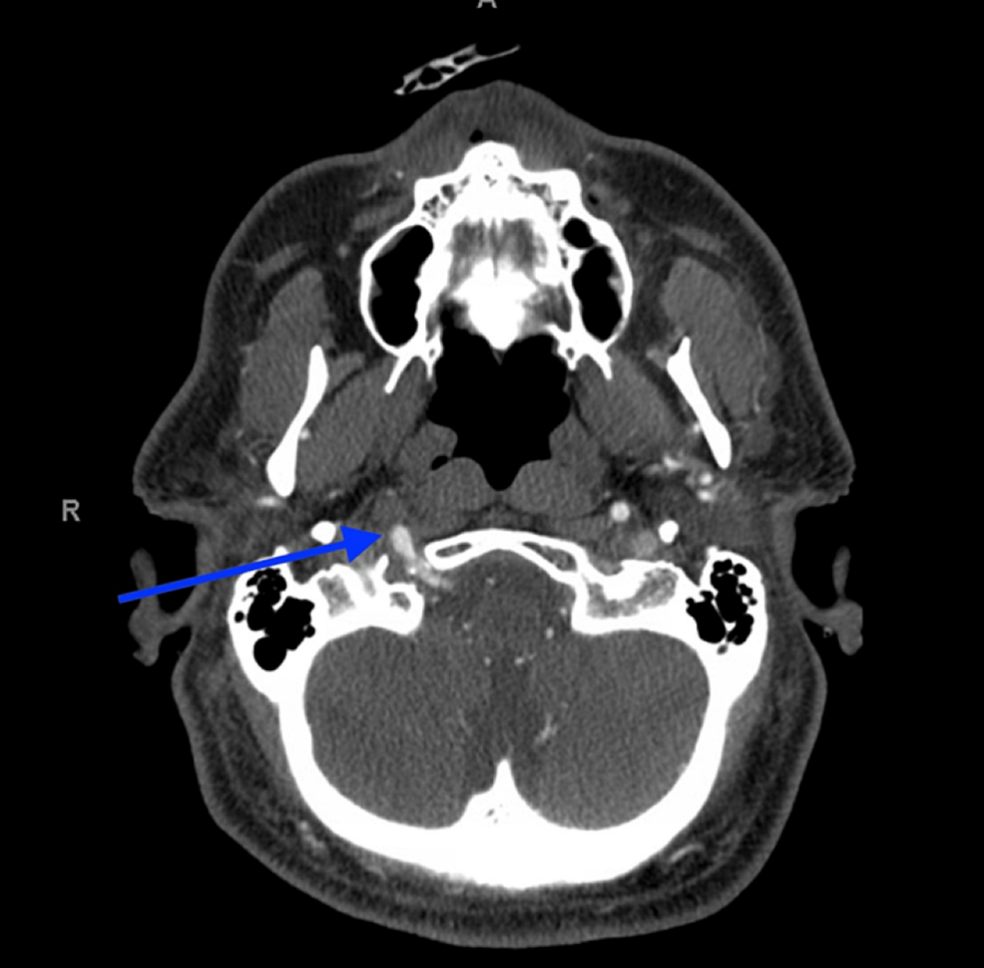
Computed Tomography Angiography Depicting the Right PPHA in Axial View

**Figure 2:**
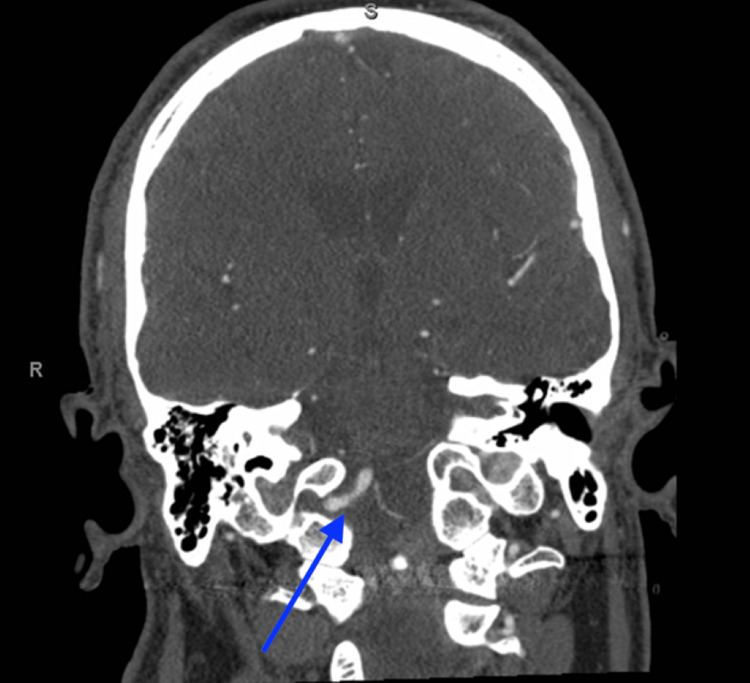
Computed Tomography Angiography Depicting the Right PPHA in Coronal View

A 60-year-old male with a past medical history of hypertension, type II diabetes mellitus, and ischemic strokes with no residual deficits (mRS = 0) presented with acute right gaze deviation, left facial droop, dysarthria, and right hemiparesis. The patient was last seen normal the evening prior at 22:00. On arrival, his last known well was greater than 4.5 hours as he presented in the morning; his NIHSS was 14, and his blood pressure was 185/100. Computed tomography (CTH) revealed a chronic right parietal lobe encephalomalacia with no acute process. Computed tomography angiography (CTA) revealed an occlusion of the right ICA with the presence of a right persistent primitive hypoglossal artery (Figures 1, 2). Alteplase was not administered as the patient was out of the window for chemical thrombolysis, but as computed tomography perfusion (CTP) revealed small core infarcts and areas of ischemic penumbra, the patient was emergently taken to the operating room for endovascular thrombectomy. Digital subtraction angiography was utilized to visualize occlusion of the right common carotid artery with no residual right internal carotid artery filling. The right PPHA was visualized with evidence of a significant thrombus along the right vertebral artery at its origin off the right internal carotid. Bobby balloon catheter was used to aspirate the thrombus in the right internal carotid artery with subsequent thrombolysis in cerebral infarction (TICI) 3 perfusion. The catheter was then advanced into the V4 segment of the right internal vertebral artery to aspirate the visualized thrombus. A Berenstein catheter was used to inject contrast, revealing full recanalization of the right vertebral artery with TICI 3 perfusion (Videos 1, 2). The patient was observed in the Neuro Critical Care Unit until stabilization. Magnetic resonance imaging (MRI) acquired on day two of admission revealed acute infracts in the right centrum semiovale, corona radiata, sub-insular cortex, temporal lobe, posterior parietal lobe, and cerebellum. The patient was discharged one week later with minor residual neurological deficits consisting of 2/5 motor deficits in the right upper and lower extremities. His right gaze deviation, left facial droop, and dysarthria had completely resolved.

**Video 1:** Digital Subtraction Angiography of PPHA in Sagittal View Arterial Phase

**Video 2:** Digital Subtraction Angiography of PPHA in Sagittal View: Arterial and Venous Phase

## Discussion

With the increased utilization of digital subtraction angiography and neuro-endovascular interventions, clinicians are encountering more anatomical variants once thought to be rare. One such variant, resulting in persistent carotid-vertebrobasilar anastomoses, is a persistent primitive hypoglossal artery. Its presence has been estimated to be 0.03% to 0.09% with unclear clinical significance, although some case reports have identified cerebral ischemia and aneurysms presenting secondary to an underlying PPHA [4,5]. It is known that the anterior and posterior circulations of the brain are two separate entities, one fed by the internal carotid artery and the other by the vertebral arteries. These distinct vascular territories assist clinicians in making diagnostic decisions, particularly in acute stroke care [6].

The neurological examination has long been a staple of localization, triage, and stratification. In this case, our patient presented with acute right gaze deviation, left facial droop, dysarthria, and right hemiparesis, making localization with a physical exam quite difficult, particularly in a specific vascular territory. The patient’s hemiparesis did not fit with his gaze deviation, which was further clouded by facial droop on the alternative side. This was a diagnostic challenge for the physicians who were assessing the patient before imaging studies. Subsequently, a digital subtraction angiography (DSA) of the head and neck was acquired, revealing an occlusion of the right ICA and the presence of the right persistent primitive hypoglossal artery, as depicted in Videos 1, 2. Fortunately, after the endarterectomy, the patient had a good outcome and was discharged from the hospital with minor neurological deficits. His MRI during admission revealed scattered areas of minor infarcts, further stressing the importance of endarterectomy in aborting what would have resulted in a much larger infarct with long-lasting detrimental neurological deficits. In this case, we would like to stress the importance of familiarizing oneself with neurovascular anatomy while also considering anatomical variants, as infarcts involving such aberrant vessels can result in a peculiar neurological examination.

## Conclusions

The patient’s presenting symptoms of right-sided gaze deviation, left facial droop, dysarthria, and right hemiparesis yielded a unique neurological examination. On initial evaluation, it was difficult to localize a solitary lesion, which would account for the patient’s acute presentation. In retrospect, with angiography revealing a right PPHA, his presentation fit more thoroughly with the clinical picture. It is important to note that with the increased utility of digital subtraction angiography and neuro-endovascular procedures, clinicians have a higher probability of encountering diverse angiographical findings. With this case, we would like to familiarize practitioners with the presence of PPHA, present unique imaging findings involving typically isolated vascular territories, and stress the importance of clinical judgment when making decisions regarding stroke care.
